# *De novo* transcriptome assembly, development of EST-SSR markers and population genetic analyses for the desert biomass willow, *Salix psammophila*

**DOI:** 10.1038/srep39591

**Published:** 2016-12-20

**Authors:** Huixia Jia, Haifeng Yang, Pei Sun, Jianbo Li, Jin Zhang, Yinghua Guo, Xiaojiao Han, Guosheng Zhang, Mengzhu Lu, Jianjun Hu

**Affiliations:** 1 State Key Laboratory of Tree Genetics and Breeding, Key Laboratory of Tree Breeding and Cultivation of the State Forestry Administration, Research Institute of Forestry, Chinese Academy of Forestry, Beijing, 100091, China; 2 Collaborative Innovation Center of Sustainable Forestry in Southern China, Nanjing Forestry University, Nanjing, 210037, China; 3 College of Forestry, Inner Mongolia Agricultural University, Hohhot, 010019, China

## Abstract

*Salix psammophila*, a sandy shrub known as desert willow, is regarded as a potential biomass feedstock and plays an important role in maintaining local ecosystems. However, a lack of genomic data and efficient molecular markers limit the study of its population evolution and genetic breeding. In this study, chromosome counts, flow cytometry and SSR analyses indicated that *S. psammophila* is tetraploid. A total of 6,346 EST-SSRs were detected based on 71,458 *de novo* assembled unigenes from transcriptome data. Twenty-seven EST-SSR markers were developed to evaluate the genetic diversity and population structure of *S. psammophila* from eight natural populations in Northern China. High levels of genetic diversity (mean 10.63 alleles per locus; mean H_E_ 0.689) were dectected in *S. psammophila*. The weak population structure and little genetic differentiation (pairwise *F*_*ST*_ = 0.006–0.016) were found among Population 1-Population 7 (Pop1-Pop7; Inner Mongolia and Shaanxi), but Pop8 (Ningxia) was clearly separated from Pop1-Pop7 and moderate differentiation (pairwise *F*_*ST*_ = 0.045–0.055) was detected between them, which may be influenced by local habitat conditions. Molecular variance analyses indicated that most of the genetic variation (94.27%) existed within populations. These results provide valuable genetic informations for natural resource conservation and breeding programme optimisation of *S. psammophila*.

Willows (*Salix*; Salicaceae) are dioecious catkin-bearing plants that are mainly distributed in the northern hemisphere. Worldwide, there are approximately 330–500 species, with various growth habits ranging from tall trees, through shrubs and bushes, to prostrate, dwarf, and rockery plants^1^. The basic chromosome number of the *Salix* genus is 19, and the ploidy levels range from diploid (2n = 2x = 38) to dodecaploid (2n = 12x = 228), with 40% of polyploid species being tetraploid to octoploid^1^,^2^,^3^,^4^. Polyploidy plays an important role in the evolution of natural populations and alters plant morphology, phenology, physiology and/or ecology within only one or a few generations and contributes to the generation of new and improved species, which benefits breeding improvement^5^,^6^. *Salix miyabeana* is a tetraploid species that has been hybridised with diploid species in recent breeding to produce triploid progeny with superior performance. An investigation of its interspecific hybrid progeny found that the triploid progeny had higher yields and better overall performance than did the diploid or tetraploid progeny^7^. Therefore, research into ploidy levels can aid taxonomic and evolutionary studies, and may accelerate the breeding process of willows.

*Salix psammophila* is an important desert shrub willow that has extraordinary adaptation to abiotic stress. It is distributed in the sandy soil in Northern China, including the Kubuqi desert and Mu Us sandland in Inner Mongolia, Yulin in Shaanxi Province, Yanchi in Ningxia Province, and others. It is planted to prevent wind erosion and to control desertification, and has played a significant role in local vegetation rehabilitation^7^. Moreover, *S. psammophila* is regarded as a promising biomass feedstock for biofuels and bioenergy because of its ease of vegetative propagation, minimal fertiliser requirement and substantial biomass. The stems of *S. psammophila* require flat stubble once every 3–5 years to allow growth to flourish and its branch residues contain high cellulose, hemicellulose and lignin contents of 55.45%, 18.98% and 25.98%, respectively^8^,^9^. The cellulose content of *S. psammophila* is higher than those reported in the study of Serapligia *et al*.^10^, which included 75 diploid, triploid and tetraploid shrub willows with cellulose content ranging from 39.9% to 46.64%.

Despite its remarkable advantages and value, few studies on *S. psammophila* have been performed. The ploidy level of *S. psammophila* remains unknown. The origin, evolution and genetic breeding of *S. psammophila* are constrained by the lack of abundant efficient molecular markers. Molecular markers have great potential to speed up the breeding process and have wide applications in cultivar identification, genetic diversity analyses, marker trait association and quantitative trait locus mapping^11^,^12^,^13^. Among the various types of markers, microsatellites or simple sequence repeats (SSRs), with tandem repeats of 2–6 nucleotide motifs, are regarded as very useful markers because of their advantages of co-dominance, stability, multi allelism, high reproducibility, chromosome specificity and ease of detection^14^,^15^,^16^.

To date, some polymorphic SSR markers have been developed in *S. reinii*^17^, *S. burjatica*^18^, *S. viminalis*^19^, *S. hukaoana*^20^, *S. arbutifolia*^21^, *S. humboldtiana*^22^, and three sub-arctic willow species (*S. lanata, S. lapponum* and *S. herbacea*)^23^. Based on these developed SSR markers, the reproduction dynamics, genetic diversity, population structure and gene flow have been investigated in several willow species. For example, both clonal growth and seedling recruitment are involved in the reproduction dynamics of *S. reinii* on the southeastern slope of Mount Fuji^24^. Sexual reproduction is predominant in *S. lanata* and *S. lapponum* at Coirre Sharroch of the Glen Clova Mountains, whereas clonal growth plays an important role in *S. herbacea* at Meall Ghaordie^25^. The levels of genetic differentiation among naturalised *S. purpurea* populations are higher than that among native *S. eriocephala* populations^26^. High levels of genetic diversity have been revealed in natural populations of *S. daphnoides* at the westernmost foothills of the Carpathian Mountains^27^ and *S. viminalis* in the Czech Republic and Europe^28^,^29^. Moreover, high levels of gene flow and genetic diversity of *S. caprea* have been detected in semi-natural woodlands across Ireland^30^. However, the study of molecular markers and population evolution of *S. psammophila* has received little attention. Thus, to speed up its marker-assisted selection breeding programmes, developing species-specific SSR markers for examining the genetic diversity and population structure of *S. psammophila* is essential.

Currently, research into *S. psammophila* is still at an early stage and the development of SSR markers is limited by the lack of genomic and transcriptomic information. In recent years, Illumina high-throughput sequencing technology has emerged as a powerful tool for obtaining genomic or transcriptomic data^31^. With the aid of this advanced technology, abundant transcript sequences have been obtained for many non-model plants (e.g., *Cryptomeria japonica, Coffea arabica, Vigna radiate, Lavandula, Caragana korshinskii* and *Phaseolus vulgaris*), and ample expressed sequence tag (EST)-SSR markers have been developed from the transcript sequences^32^,^33^,^34^,^35^,^36^. These studies indicate that it is efficient and feasible to mine microsatellites and develop markers using high-throughput transcriptome sequencing.

In this study, we performed chromosome counts and genome size estimation of *S. psammophila*, reported the first transcriptome sequencing from various tissues of *S. psammophila*, developed EST-SSR markers based on the transcriptome sequencing data, and analysed the genetic diversity and population structure of eight natural populations in Northern China. This work provides fundamental information for natural resource conservation and breeding programme optimisation of *S. psammophila* in the future.

## Results

### Chromosome counts and genome size estimation of *S. psammophila*

**Figure 1:**
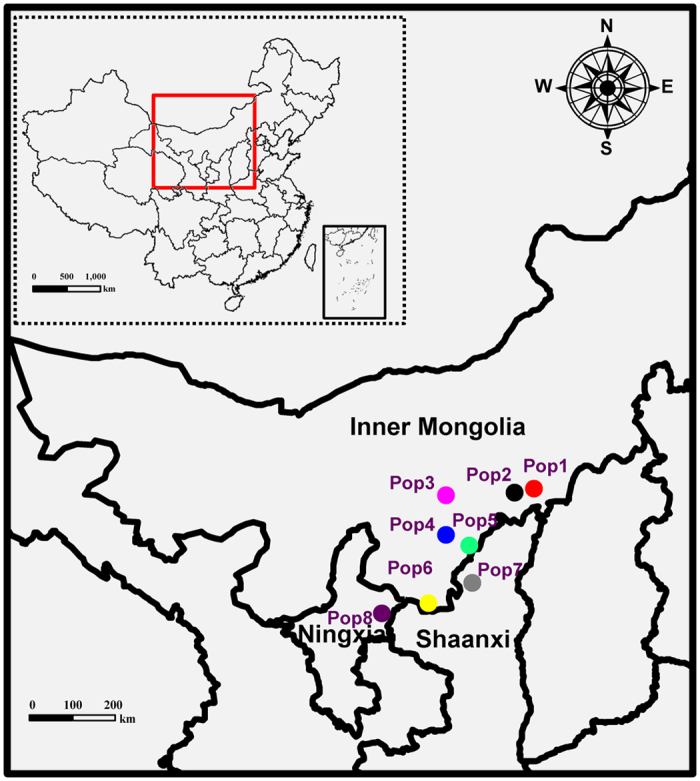
Locations of the eight natural populations of *S. psammophila*. The map was created using ESRI ArcGIS 10.1 software. *Scientific Reports* remains neutral with regard to contested jurisdictional claims in published maps.

**Figure 2:**
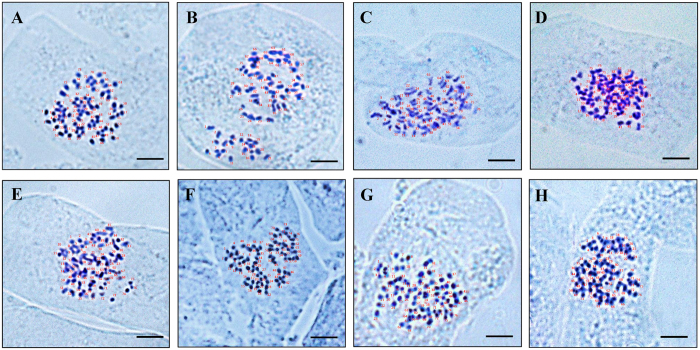
Somatic chromosomes in root meristem cells of *S. psammophila*. (**A**–**H**) A total of eight genets were selected. One genet was selected from each of the eight natural populations (from Pop1 to Pop8). Scale bar = 5 μm.

To reveal the ploidy level of *S. psammophila*, eight genets from different populations that represented the main distribution areas, including six populations from Inner Mongolia, one population from Shaanxi Province and one population from Ningxia Province (Fig. 1, Table 1), were used for chromosome counts in root tip squashes. As shown in Fig. 2, the eight different genets had the same chromosome numbers, for 2n = 4x = 76. This indicated that *S. psammophila* is naturally tetraploid. Flow cytometry (FCM) technique was performed to estimate the genome size using the fresh leaves of 27 genets, including the eight genets used for chromosome counts and 19 additional genets. With its genome size of approximately 425–429 Mb, *S. suchowensis*^37^ served as the internal reference standard. The ratio of the G1 peak means of *S. psammophila* to that of *S. suchowensis* was equal to 1.645, indicating that the genome size of *S. psammophila* is estimated to be approximately 699–706 Mb (Fig. S1, Table S1).

### Illumina sequencing, *de novo* assembly and functional annotation of unigenes

Equal quantities of RNA from five tissues including the root, stem, leaf, female catkin and male catkin of *S. psammophila* were mixed and used to construct a cDNA library for sequencing based on the Illumina HiSeq2500 platform. A total of 45,600,129 raw reads with paired-ends of 125 bp were obtained. After quality control, 44,343,202 clean reads (11.17 Gb) were retained. The GC content was 45.28%, and the Q20 and Q30 were 94.73% and 90.55%, respectively. With the aid of the short-reads assembling software Trinity, these clean reads were *de novo* assembled into 159,737 contigs, and then into 71,458 non-redundant unigenes, with an N50 length of 1,274 bp and a mean length of 713 bp (Fig. S2). The sequencing data have been deposited in the NCBI Sequence Read Archive (http://www.ncbi.nlm.nih.gov/Traces/sra) under accession number SRA3189819.

**Figure 3:**
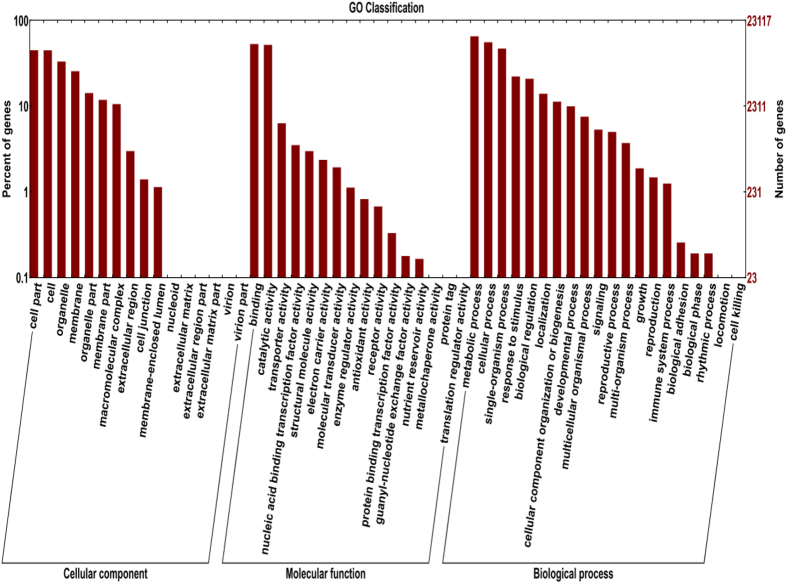
Gene ontology (GO) classification of *S. psammophila* unigenes.

**Figure 4:**
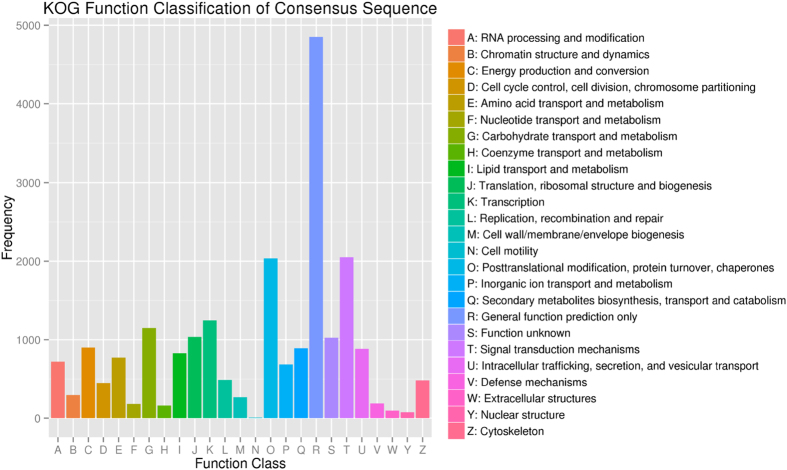
Eukaryotic orthologous groups (KOG) classification of *S. psammophila* unigenes.

To annotate the unigenes of *S. psammophila* using a bioinformatics approach, a sequence similarity search was conducted in six public databases (Table 2). The results showed that 37,151 (51.99%) unigenes had significant matches in the non-redundant protein sequences (Nr) database; 21,556 (30.17%) had significant matches in the protein family (Pfam) database; 24,277 (33.97%) had significant matches in the Swiss-Prot database; 23,117 (32.35%) had significant matches in the Gene Ontology (GO) database; 19,425 (27.18%) had significant matches in the eukaryotic orthologue groups (KOG) database; and 7,268 (10.17%) had significant matches in the Kyoto Encyclopedia of Genes and Genomes (KEGG) database. A total of 37,465 (52.43%) unigenes were annotated in at least one of these databases. In the GO analyses (Fig. 3, Table S2), 23,117 unigenes were classified into three classes, including cellular component (12,607 unigenes), molecular function (19,102 unigenes), and biological process (17,985 unigenes). There were 19,425 unigenes assigned to KOG classifications, which were divided into 25 specific categories (Fig. 4, Table S3). The category of general functional prediction, which is associated with only basic physiological and metabolic functions, was the largest group, with 4,849 unigenes (22.28%); whereas the category, cell motility, was the smallest group, with only seven unigenes (0.03%). The KEGG pathway-based analyses is helpful for understanding the biological functions and interactions of genes. A total of 7,268 unigenes had significant matches in the KEGG database and were assigned to 116 biological pathways (Table S4). The pathway with the most annotated genes was ribosome (ko03010, 442 unigenes, 5.61%), followed by plant hormone signal transduction (ko04075, 271 unigenes, 3.44%), oxidative phosphorylation (ko00190, 255 unigenes, 3.24%), protein processing in endoplasmic reticulum (ko04141, 234 unigenes, 2.97%), spliceosome (ko03040, 214 unigenes, 2.72%), glycolysis/gluconeogenesis (ko00010, 209 unigenes, 2.65%) and RNA transport (ko03013, 205 unigenes, 2.60%).

### Frequency and distribution of EST-SSRs in *S. psammophila*

All the unigenes were used to detect EST-SSRs. Of the 71,458 (50.97 Mb) unigenes of *S. psammophila*, 5,616 (7.86%) were determined to contain 6,346 SSRs. Among these developed SSRs, 424 (7.55%) contained two or more SSRs, and 403 (6.35%) comprised compound SSRs (Table 3). The frequency of EST-SSRs was one EST-SSR in 11.26 unigenes. The di-nucleotide motif was the most abundant (53.72%), followed by the tri-nucleotide (42.94%), tetra-nucleotide (2.77%), hexa-nucleotide (0.39%) and penta-nucleotide (0.17%) motifs (Table 3). There was a large proportion of both di- and tri-nucleotides (96.66%), whilst the rest amounted to 3.34%. In total, 127 different SSR motifs were identified. Among them, the di-, tri-, tetra-, penta- and hexa-nucleotide repeats were of 6, 31, 54, 11 and 25 types, respectively (Table S5). The most abundant type was AG/CT (1,048, 16.51%), followed by GA/TC (1,024, 16.14%), AT/TA (878, 13.84%), GAA/TTC (274, 4.32%), AGA/TCT (225, 3.55%), AC/GT (220, 3.47%), AAG/CTT (215, 3.39%) and CA/TG (214, 3.37%) motifs. The remaining motifs accounted for 35.42%. The AG/CT repeat was the most abundant di-nucleotide motif, and the GAA/TTC repeat was the most abundant tri-nucleotide motif. Furthermore, there were few CG/GC and CCG/CGG motifs in *S. psammophila*, with 25 CG/GC and 12 CCG/CGG motifs. Additionally, based on the length of the SSR sequence, the microsatellites were grouped into two classes: class I (12 ≤ n ≤ 20 nucleotides) and class II (n ≥ 20 nucleotides). As shown in Table S6, most SSR repeats were distributed in class I, accounting for 89.41% (5,674 SSRs) of the total SSRs, whilst 10.59% (672 SSRs) had longer repeat sequences and were distributed in class II.

### Genetic diversity of EST-SSR loci

A total of 168 EST-SSR markers containing di- and tri-nucleotide repeats were randomly selected and primers were designed according to their flanking sequences. Of the 168 EST-SSR primers, 125 pairs showed successful amplification. Finally, 27 pairs that showed stable amplification and had multiple alleles were selected to analyse the genetic diversity and population structure of 240* S. psammophila* genets from eight natural populations in Northern China (Table 1). These 27 loci were verified to be equally suited for an MAC–PR (microsatellite DNA allele counting–peak ratios) approach which calculated the ratios between the peak areas for two alleles in all samples where these two alleles occurred together (Table S7). A summary of the genetic characteristics of *S. psammophila* based on 27 SSR markers was shown in Table 4. In total, 287 alleles were amplified across the genets, with a mean of 10.63 observed alleles per locus. The expected heterozygosity (H_E_) and the Shannon–Wiener index ranged from 0.436 to 0.883 and from 0.696 to 2.133, with averages of 0.689 and 1.345, respectively. The polymorphism information content (PIC) was in the range of 0.386–0.888, with an average of 0.714. Markers c-59 and c-25 shared the highest and lowest genetic diversity, respectively, with values of 0.883 and 0.436 for H_E_ and 0.888 and 0.386 for the PIC.

Using these 27 EST-SSR makers, all 240 genets amplified a maximum of four alleles at some loci. Due to co-dominance, SSR markers present different alleles if they are heterozygous. Thus, SSR analyses also indicate that *S. psammophila* is tetraploid in the natural population.

### Population structure, principal coordinate analyses (PCoA) and neighbour joining (NJ) phylogenetic analyses

**Figure 5:**
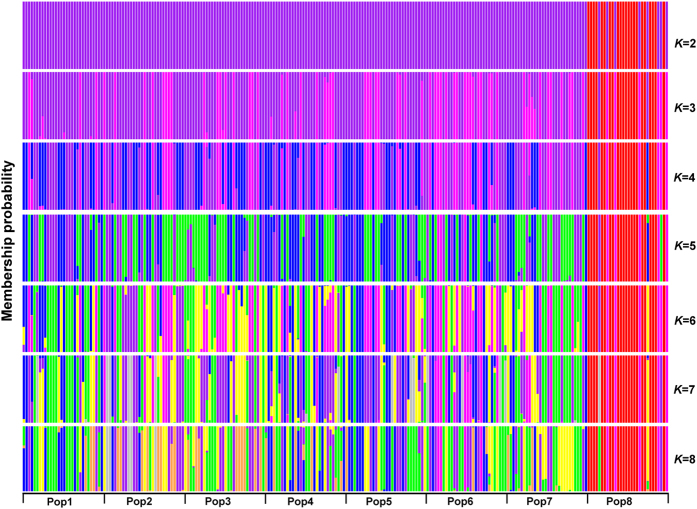
Discriminant analysis of principal components (DAPC) of the 240 *S. psammophila* genets. Cluster membership probabilities of each genet based on the discriminant functions of DAPC. The analysis was run for 2 ≤ k ≤ 8. Each genet is represented by a vertical bar.

Discriminant analyses of principal components (DAPC)^38^ was used to analyse the genetic structure for 240 *S. psammophila* genets. The optimal number of PCs (45) was assessed using the *optim.a.score* function. The cluster membership probabilities of each genet based on the discriminant functions of DAPC were performed from *K* = 2 to *K* = 8 (Fig. 5). Most genets (22 of 30) of Pop8 were separated from the other populations at *K* = 2 (Fig. 5, Fig. S3). With the increase in cluster number, population subdivision was gradually generated among Pop1-Pop7. At *K* = 8, 22 genets of Pop8 were also retained in one cluster, five genets of Pop8 and four genets of Pop6 were assigned to another cluster; and remaining genets were interspersed among six other clusters (Fig. S4).

**Figure 6:**
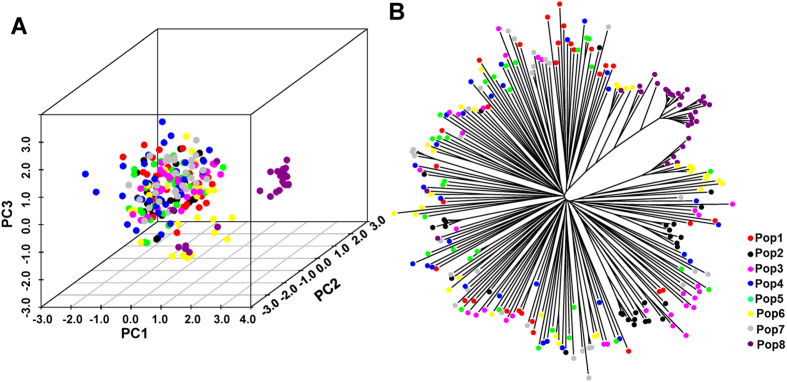
Three-dimensional principal coordinate analysis (PCoA) and neighbour-joining (NJ) tree of the 240 *S. psammophila* genets.

PCoA was conducted and NJ phylogenetic trees were constructed to further assess the population genetic structure. The first three principal coordinates explained 14.37%, 11.23% and 7.64% individually, and explained 33.24% of the total variation (Fig. 6A). The Nei’s genetic distances (GDs) of the 240 *S. psammophila* genets were calculated, which ranged from 0.002 to 0.602, with an average of 0.387 (Table S8). Based on the GDs, an NJ tree was constructed (Fig. 6B). The results of PCoA and the NJ phylogenetic tree were consistent with the DAPC analyses. Most genets of Pop8 (Ningxia) had relatively far genetic distances from other populations, and some genets of Pop6 were classified into the Pop8 group.

**Figure 7:**
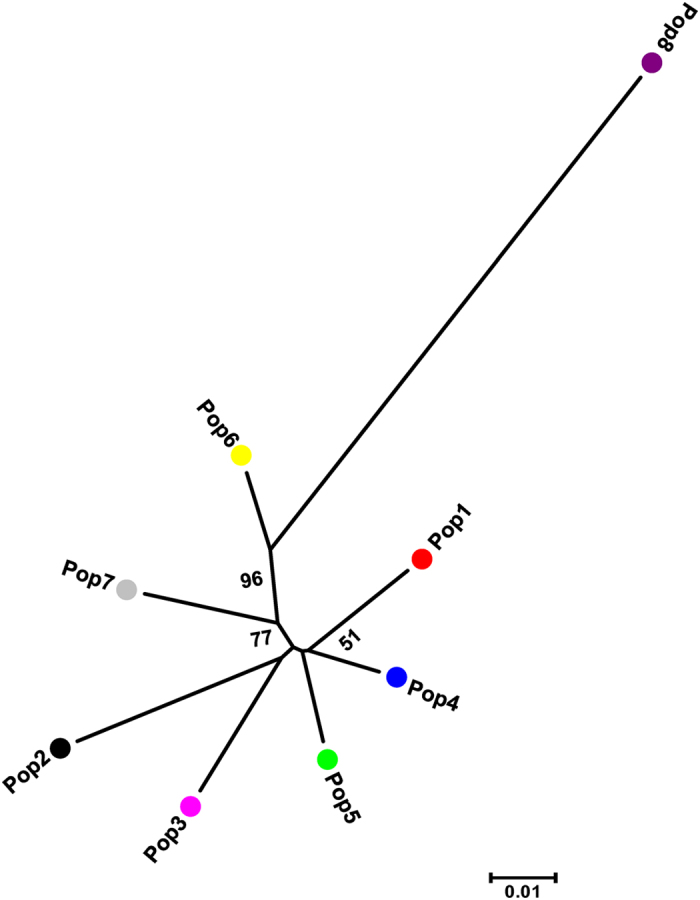
Neighbour-joining (NJ) tree based on Nei’s genetic distances for the eight *S. psammophila* populations. Node values above the branches indicate bootstrap support percentage over 50% in 1000 pseudoreplicates.

Furthermore, the GDs of the populations and the NJ phylogenetic tree were also used to evaluate the genetic relationships of the eight populations. As shown in Table 5 and Fig. 7, the GDs among Pop1 to Pop7 were 0.028–0.068, and Pop4 was genetically more closely related to Pop5, with a GD 0.028, followed by Pop1 being closer to Pop4 and Pop5, with GDs of 0.033 and 0.038, respectively. The GDs of Pop8 from the other population were larger than the other combinations, ranging from 0.112 to 0.151, which indicate that the genets from Ningxia (Pop8) have a distant genetic relationship with the other seven populations. In particular, the genetic relationship between Pop3 and Pop8 was the farthest (GD 0.151).

### Population differentiation and genetic diversity

To analyse the genetic differentiation among the eight populations, pairwise Wright’s *F*_*ST*_ was calculated using the “polysat” software in R package. The lowest genetic differentiation was detected between Pop4 and Pop5 (*F*_*ST*_ = 0.006), and the greatest genetic differentiation was detected between Pop3 and Pop8 (*F*_*ST*_ = 0.055) (Table 5). The genetic differentiation among Pop1-Pop7 was generally low (average pairwise *F*_*ST*_ = 0.011, range 0.006–0.016), whilst the genetic differentiation between Pop8 and Pop1-Pop7 was relatively high (average pairwise *F*_*ST*_ = 0.050, range 0.045–0.055). The genetic differentiation between Pop8 and Pop6 (*F*_*ST*_ = 0.045) was slightly lower than that between Pop8 and the six other populations (Table 5).

The genetic diversity of the eight populations was assessed, and the result was shown in Table 6. Pop8 (Ningxia) had the lowest value for alleles per locus, H_E_, Shannon-Wiener index and PIC, suggesting that the genets from Ningxia have low genetic diversity compared with those from the Inner Mongolia and Shaanxi populations. Obvious differences in genetic diversity between Pop8 and the other seven populations were detected.

Analyses of molecular variance (AMOVA) indicated that 5.73% of the total molecular variance resided among eight populations, whilst 94.27% was attributed to variance within populations (Table 7), suggesting that higher variation existes within populations than among populations of *S. psammophila*. To test the proportion of genetic variance between clusters with the *K* = 2 suggested by the DAPC analyses, AMOVA was performed and showed that 18.56% of the total molecular variance was attributed to between two clusters (Table 7). Though the higher variation existed within clusters than among clusters, this variance proportion among clusters was higher than that among populations.

## Discussion

The *Salix* genus comprises many species, among which polyploids are common^1^,^2^,^3^,^4^. The approaches used for ploidy detection mainly include chromosome counts, FCM technique, and SSR analyses^2^,^39^. In this study, *S. psammophila* was found to be tetraploid by chromosome counts. Second, using FCM technique, the G1 peak means of 19 additional genets were similar to that of the eight tetraploid genets, suggesting that these 19 genets are also tetraploids. The genome size of *S. psammophila* was estimated to be approximately 699–706 Mb, which is approximately 1.6-fold than *S. suchowensis* with approximately 425–429 Mb. Previous studies of soybeans have shown that the genome size of the allotetraploid soybean (*Glycine dolichocarpa*) is approximately 1.9-fold than its diploid progenitor (*G. tomentella* and *G. syndetika*) and the tetraploid retains at least approximately 87% of the genes that were initially duplicated^40^. In *Knautia*, the results of ploidy and genome size of 23 species indicated that genome size of tetraploid *K. arvernensis* and hexaploid *K. ressmannii* is approximately 1.8-fold and approximately 2.7-fold than diploid *K. calycina*, respectively^41^. Similar results of the genome size being inconsistent with the ploidy level were also observed in *Chenopodium*^42^ and *Avena*^43^. One likely reason for this phenomenon is that some genes or chromosome fragments with functional redundancy had not doubled in a genome duplication event, or they were deleted after genome duplication event^44^. Finally, all 240 *S. psammophila* genets presented a maximum of four alleles at some loci in the SSR analyses, indicating that these genets are tetraploids. Multiple alleles were also detected in *S. reinii* using SSR markers, suggesting that *S. reinii* is polyploid; this inference was confirmed by the FCM technique^17^,^24^. These results indicate that SSR marker analyses is a simple and efficient method for determining ploidy. Furthermore, our study did not find the variability of ploidy level in 240 *S. psammophila* genets. Other ploidy levels (i.e., 2x, 3x, 5x, 6x, etc.) of *S. psammophila* may exist in natural populations, and these materials are more meaningful for the study of genome evolution. Therefore, the intraspecific ploidy variation of *S. psammophila* requires further research.

Some hypotheses suggest that polyploids have greater resistance to harsh arctic climates than diploids, or that polyploids are more successful than diploids in colonising after deglaciation^45^. In the Helix section, compared with other diploid willow species (*S. suchowensis* and *S. purpurea*)^37^, *S. psammophila* was verified to be tetraploid and mainly distributed in the desert regions in Northern China. Therefore, the tetraploid nature of *S. psammophila* may be associated with strong adaptability to infertile soil and harsh climatic environment. Moreover, polyploids have been reported to affect the gene expression at the transcription level compared with diploids. For example, studies in watermelon have shown that 5,362 and 1,288 genes were up- and down-regulated, respectively, in autotetraploid compared with diploid watermelon^46^. So further studies are required to reveal the reason for tetraploid formation and the effect on gene expression after genome duplication event in *S. psammophila*.

Recently, willows have become a focus of research as promising sources for bioenergy crops because of their short rotation coppice cycle and high biomass yields^1^,^47^. The full genome sequences of *S. suchowensis* (http://115.29.234.170/willow/)^37^ and *S. purpurea* (http://phytozome.jgi.doe.gov/pz/portal.html#) have been released in public databases. In addition, transcriptome analyses of *S. matsudana* and *S. viminalis* have been performed to reveal their stress response mechanisms^48^,^49^. However, no genomic or transcriptomic data have been reported for *S. psammophila*. In our study, 71,458 non-redundant unigenes with a mean length of 713 bp were *de novo* assembled, and 37,465 (52.43%) unigenes were aligned to the sequences of six public databases. Based on these unigenes, 6,346 SSR loci were detected. The AG/CT repeat was the most abundant di-nucleotide motif, which is similar to *S. babylonica, S. suchowensis, Populus* and *Eucalyptus*; however, the most abundant tri-nucleotide motif was the GAA/TTC repeat, which differs from these species (AAG/CTT for *Salix* and *Populus*; CCG/CGG for *Eucalyptus*)^50^. The presence of few CG/GC and CCG/CGG motifs in *S. psammophila* agrees with previous studies that showed that CG/GC and CCG/CGG are very rare in dicotyledonous plants but common in monocots^51^. These transcript sequences and EST-SSR loci could facilitate further investigations in marker-trait association, quantitative trait locus mapping and genetic diversity analyses in *S. psammophila*.

As with other willow species, high levels of genetic diversity were found in 240 *S. psammophila* genets from eight natural populations in Northern China. The mean number of 10.63 alleles per locus and mean H_E_ 0.689 were determined using 27 developed EST-SSR markers in *S. psammophila*, which was consistent with SSR-derived genetic diversity in *S. viminalis* (mean 6.95 alleles per locus and mean H_E_ 0.65 within 84 individuals from seven sampled sites in the Czech Republic; mean 13.46 alleles per locus and mean H_E_ 0.62 within 505 individuals from five populations in Europe)^28^,^29^ and *S. caprea* (mean 10 alleles per locus and mean H_E_ 0.58 within 183 individuals from 21 semi-natural woodlands)^30^. Although the studied genets of *S. psammophila* were sampled within a 220 kilometres radius, it is a dioecious and cross-pollination species, and its pollen can disperse over long distances due to windy weather in the pollination period and few obstacles to airborne pollen flow in desert regions, which may explain its high levels of genetic diversity. The small seeds (thousand seed weight of approximately 0.1 g) of *S. psammophila* are wrapped in fine hairs that facilitate dispersal by wind. Moreover, the tetraploid nature of this species may be another reason for the high genotype diversity of *S. psammophila*.

AMOVA indicated that most (94.3%) of the total variance was within-population variance. This agrees with the results obtained for other *Salicaceae* species, such as *S. viminalis* (92.3%)^28^, *Salix caprea* (91.7%)^30^, *Populus simonii* (85.8%)^52^, *P. euphratica* (85.5%) and *P. pruinosa* (78.8%)^53^ based on SSR markers. This result may have been obtained because *Salicaceae* species are generally dioecious and combine anemophilous and entomophilous pollination with an outcrossing breeding system and high dispersal ability, which could enhance intraspecific diversity and reduce population differentiation^27^,^54^,^55^. Additionally, the relatively small geographic range would see more frequent gene flow by pollen and seed dispersal and result in little differentiation among populations of *S. psammophila*. However, pollination dynamics and the mating system of *S. psammophila* remain to be poorly understood, and should be further studied to better understand the distribution of genetic variation.

The weak population structure and little genetic differentiation (pairwise *F*_*ST*_ = 0.006–0.016) were found among Pop1-Pop7 (Inner Mongolia and Shaanxi), but Pop8 (Ningxia) was clearly separated from Pop1-Pop7, and moderate differentiation (pairwise *F*_*ST*_ = 0.045–0.055) was detected between them. Previous studies have demonstrated that the habitat types or local environment conditions have a significant influence on the genetic diversity and population structure in some plant species, such as *Ranunculus acris*^56^, *Paris quadrifolia*^57^, *Rhododendron jinggangshanicum*^58^ and *Tamarix chinensis*^59^. In this study, the Pop1-Pop7 genets were sampled around Kubuqi desert or Mu Us Sandland with typical arid desert habitats, whilst the Pop8 genets were sampled around Haba Lake with wetland habitats. To adapt to these two different habitat conditions, the genotypes of *S. psammophila* were selected and appeared to differentiation during the long-term evolutionary process. A germplasm collection base of *S. psammophila* was established approximately 20 kilometres northeast of Pop1 in Inner Mongolia, with its habitat condition belonging to an arid desert habitat. In recent years, we found that most of Pop8 genets appeared dead due to unadaptability to the arid desert habitats in the germplasm collection base, and this phenomenon did not occur in Pop1-Pop7 genets, supporting above mentioned hypothesis. Moreover, the habitat variability could lead to different phenophases, which may constrain the gene flow between Pop8 and other populations. However, detailed reasons for the population genetic structure need to be further studied.

## Methods

### Plant materials and DNA extraction

To protect the germplasm resources and to carry out the molecular biology study of *S. psammophila*, the germplasm collection base of *S. psammophila* (E 110°38′59.1″, N 40°14′15.5″) was established in Ordos Dalad, Inner Mongolia in 2008. In total, 21 *S. psammophila* populations were collected, including Inner Mongolia (15 populations), Shaanxi (4 populations) and Ningxia (2 populations); each population contained approximately 50 samples. To avoid sampling clones, each sample was randomly selected and separated by at least 50–100 m from the next sample. Two-year-old twigs were collected and cut into 15–20 cm cuttings. Subsequently, these cuttings were planted in the germplasm collection base of *S. psammophila*. In this study, eight populations covering the main natural distribution areas were selected from the 21 populations in the germplasm collection base for population genetic analyses of *S. psammophila*, and 30 genets were randomly sampled in each population. Details of the locations of the eight populations used in this study are shown in Fig. 1 and Table 1. Fresh leaves were dried and stored in silica gel. DNA was isolated using the cetyltrimethylammonium bromide method^60^, and DNA quality and concentration were estimated using the NanoDrop system (Thermo Scientific, USA) and 1.0% agarose gel electrophoresis.

### Chromosome counts

Eight genets (one from each of the eight natural populations) were used for chromosome counts. Twigs were collected from 1-year-old seedlings of *S. psammophila* and grown in hydroponic culture in a growth chamber under long-day conditions (16 h light/8 h dark) at 25 °C. When new roots grew out, root tip squashes were used to enumerate the chromosome number according to the method described in a previous study with some modifications^61^,^62^. To obtain mitotic metaphases, root tips were pre-treated with 2 mM 8-hydroxyquinoline for approximately 2 h and washed three times with ddH_2_O. The root tips were fixed in Carnoy’s fluid (absolute ethanol:glacial acetic acid, 3:1) for 24 h at room temperature, steeped in 90% ethanol for 30 min, and preserved in 70% ethanol at 4 °C. To prepare slides, roots were hydrolysed in 1 M hydrochloric acid for 15–20 min at 60 °C. The slides were prepared according to the squash technique with carbol fuchsin. Images were captured using Zeiss Axio Imager A1 microscope. The chromosome number was determined by counting more than ten well-spread chromosomal cells from each of the eight genets.

### Flow cytometry measurements

The genome size of *S. psammophila* was estimated using flow cytometry (FACSCalibur cytometer, BD Bioscience) according to a previous protocol^63^. Fresh leaves of 27 genets, including the eight genets used for chromosome counts and 19 additional genets, were fully chopped with a razor blade in the presence of 2 ml lysis solution, filtered through a 300-μm nylon mesh, collected in 5 ml glass tube, and centrifuged for 5 min at 5000 r·min^−1^. After discarding the supernatant, the nuclei suspension was stained with 200 μl propidium iodide (PI) at 4 °C for 30 min in the dark. *S. suchowensis* (approximately 425–429 Mb)^37^ was used as an internal standard. The genome size was calculated based on the values of the G1 peak means as follows: sample genome size = [(sample G1 peak mean)/(standard G1 peak mean)] × standard genome size.

### RNA isolation, library preparation, Illumina sequencing and *de novo* transcriptome assembly

Five tissues (root, stem, leaf, female catkin and male catkin) were obtained from an elite tree of *S. psammophila*. Total RNA was extracted using the RNeasy Plant Mini Kit (Qiagen, Hilden, Germany) according to the manufacturer’s instructions. The quality and quantity of total RNA were determined by the NanoDrop system (Thermo Scientific, USA) and an Agilent2100 Bioanalyser (Agilent Technologies, USA). mRNAs were enriched with a NEBNext Poly (A) mRNA Magnetic Isolation Module (NEB, E7490). Equal quantities of enriched mRNAs of the five tissues were mixed and used as templates to synthesize cDNA. The cDNA library was constructed using the NEBNext Ultra mRNA Library Prep Master Mix Set for the Illumina (NEB, E7530) and NEBNext Multiplex Oligos for the Illumina (NEB, E7500) according to the manufacturer’s instructions. Finally, the cDNA library was sequenced on an Illumina HiSeq2500 instrument by Biomarker Technologies Co. Ltd. (Beijing, China), with paired-end sequencing technology and read lengths of 125 bp. As a part of the quality control procedure, adapter sequences and low quality reads were filtered for subsequent analyses. The resulting high quality sequences were assembled using Trinity software^64^.

### Unigene annotation and classification

For functional annotation and classification, the assembled unigenes were aligned using BLASTx alignment (E-value <1 × 10^−5^) with public databases, including the NCBI non-redundant protein sequences (Nr) database (http://www.ncbi.nlm.nih.gov/), the Swiss-Prot database (http://www.expasy.ch/sprot/), the eukaryotic orthologue groups (KOG) database (http://www.ncbi.nlm.nih.gov/COG), the Kyoto Encyclopedia of Genes and Genomes (KEGG) database (http://www.genome.jp/kegg/), and the protein family (Pfam) database. Gene Ontology (GO) annotation of these unigenes was produced using the Blast2GO program^65^ based on the results of the NCBI Nr database annotation.

### SSR loci identification and SSR markers

The obtained unigenes were used to identify potential SSRs using the MISA tool (http://pgrc.ipk-gatersleben.de/misa/). The parameters were designed to identify di-nucleotide motifs with a minimum of six repeats, and tri-, tetra-, penta-, and hexa-nucleotide motifs with a minimum of five repeats^34^. In total, 168 SSR primers were designed and synthesised by Thermo Fisher Scientific (Shanghai, China). Fluorescence-labelled TP-M13-SSR (SSR with a tailed M13 primer) analyses was performed as described by Schuelke^66^. PCR amplifications were performed in a reaction volume of 20 μl, containing 100 ng template DNA, 1×  PCR buffer (Takara, Dalian, China), 100 μM each dNTP, 30 μM MgCl_2_, 1.0 unit *Taq* DNA polymerase (Takara), 8 pmol each reverse primer and the M13 universal primer (5′-TGTAAAACGACGGCCAGT-3′) that was fluorescently labelled with Cy5 at the 5′ terminus, and 2 pmol forward primer that was added to the M13 primer sequence to the 5′ terminus. PCR amplification was performed on an ABI Applied Biosystems instrument under following conditions: 94 °C for 5 min; 30 cycles at 94 °C for 30 s, 53 °C for 30 s and 72 °C for 30 s; 8 cycles at 94 °C for 30 s, 57 °C for 30 s and 72 °C for 30 s; and a final extension step at 72 °C for 10 min. The capillary electrophoretic separation of PCR products was performed using *GenomeLab GeXP* (Beckman Coulter, USA), and the data were analysed by the fragment analyses module of the system.

### Data analyses of genetic diversity and population structure

Using the MAC-PR method, all alleles of each locus were analysed in pairwise combinations to determine their dosages in the genets by calculating the ratios between peak areas^67^. The expected heterozygosity and Shannon-Wiener diversity indices were calculated using ATETRA version 1.2 (http://www.vub.ac.be/APNA/)^68^. The PIC of the SSR markers was calculated using PIC_CALC version 0.6 as described by Botstein *et al*.^69^. MI, as an overall measure of the efficiency to detect polymorphisms, was calculated as described by Powell *et al*.^70^. The genetic differentiation index (Wright’s *F*_*ST*_) was calculated using “polysat” software^71^ in the R x64 3.2.3 package. DAPC^38^ from the *adegenet* package version 2.0.1^72^ in the R x64 3.2.3 package was used to analyse the genetic structure. This method does not need to take into account some assumptions about Hardy-Weinberg equilibrium and linkage disequilibrium. DAPC requires enough PCs to secure a space with sufficient power of discrimination but must also avoid retaining too many dimensions that lead to over-fitting^38^. Therefore, we chose the optimal number of principal components for DAPC using the *optim.a.score* function. The genetic distances between the genets and populations were calculated using Populations version 1.2.31 (http://www.bioinformatics.org/~tryphon/populations/) according to the method described by Nei *et al*.^73^. The genetic distance matrix was used to construct a dendrogram using the NJ method in MEGA version 6.06^74^. Further analyses of the genetic structure was performed by PCoA using GenAlEx version 6.5^75^, and a tri-scatter plot was generated using the R x64 3.2.3 package. To estimate the variance component and to partition the variation within and among populations and clusters, AMOVA was performed using GenAlEx^75^.

## Additional Information

**How to cite this article**: Jia, H. *et al. De novo* transcriptome assembly, development of EST-SSR markers and population genetic analyses for the desert biomass willow, *Salix psammophila. Sci. Rep.* **6**, 39591; doi: 10.1038/srep39591 (2016).

**Publisher's note:** Springer Nature remains neutral with regard to jurisdictional claims in published maps and institutional affiliations.

## Supplementary Material

Supplementary Information

Supplementary Dataset 1

Supplementary Dataset 2

Supplementary Dataset 3

Supplementary Dataset 4

Supplementary Dataset 5

Supplementary Dataset 6

Supplementary Dataset 7

Supplementary Dataset 8

## Figures and Tables

**Table 1:** Location and sampling site characteristics for the eight *S. psammophila* populations.

Populations	Location	Number	Longitude (E)	Latitude (N)	Altitude/m
Pop1	Juhetan, Zhungeer, Inner Mongolia	30	111°00′	40°11′	1074
Pop2	Wulanhao, Dalate, Inner Mongolia	30	110°35′	40°04′	1217
Pop3	Wuritu, Hangjin, Inner Mongolia	30	108°49′	40°00′	1436
Pop4	MuKaizhuoer, Etuoke, Inner Mongolia	30	108°47′	39°16′	1180
Pop5	Tuke, Wushen, Inner Mongolia	30	109°22′	39°03′	1325
Pop6	Chengchuan, Etuoke, Inner Mongolia	30	108°18′	37°39′	1198
Pop7	Qiaojiamao, Yulin, Shaanxi	30	109°24′	38°11′	1158
Pop8	Haba Lake, Yanchi, Ningxia	30	107°03′	37°42′	1468

**Table 2:** Summary of functional annotation of assembled unigenes of *S. psammophila*.

Annotation_Database	Number of unigenes	Percentage (%)
Annotated in Nr	37,151	51.99
Annotated in Pfam	21,556	30.17
Annotated in Swiss-Prot	24,277	33.97
Annotated in GO	23,117	32.35
Annotated in KOG	19,425	27.18
Annotated in KEGG	7,268	10.17
Annotated in at least one Database	37,465	52.43
Total unigenes	71,458	100.00

**Table 3:** Summary of EST-SSRs found in *S. psammophila*.

Searching items	Numbers
Total number of sequences examined	71,458
Total size of examined sequences (bp)	50,966,464
Total number of identified SSRs	6,346
Number of sequences containing SSRs	5,616
Number of sequences containing more than 1 SSR	424
Number of SSRs present in compound formation	403
Di-nucleotide	3,409
Tri-nucleotide	2,725
Tetra-nucleotide	176
Penta-nucleotide	11
Hexa-nucleotide	25

**Table 4:** Amplification results of 27 EST-SSR primers in *S. psammophila*.

SSR name	Gene ID	Motifs	Primer sequences (forward and reverse)	Length of fragment (bp)	No. of alleles	No. of poly- morphic alleles	No. of alleles per genet	Expected heterozygosity (H_E_)	Shannon-Wiener index	Polymorphism information content (PIC)	Marker index (MI)
c-4	c25926.graph_c0	(AG)_8_	F: CTTCCACATGCCTCTGACAA	242–266	11	11	2.58	0.711	1.401	0.789	8.679
			R: TTGGACACAGACACGCTTTT								
c-13	c41813.graph_c0	(CT)_8_	F: CGGCCTAACAATCTAAGCCA	113–153	13	13	2.43	0.736	1.552	0.736	9.572
			R: CATGGCAGCTTCACAGATTG								
c-16	c43032.graph_c0	(TTC)_5_	F: CTTCTCGGCTTCAACTTTCG	211–238	7	7	2.21	0.547	1.061	0.629	4.404
			R: ACAATTCCAATAACCCGCAG								
c-24	c45977.graph_c0	(GT)_8_	F: ATGGAGATCAGCAGTGAGCC	257–287	14	14	2.75	0.764	1.575	0.798	11.168
			R: TTGCTCTGGGGATTTTCTTG								
c-25	c45986.graph_c0	(TG)_6_	F: TTCACGTCCTCTCTTTGCCT	186–192	4	3	1.73	0.436	0.696	0.386	1.158
			R: CCTCTAGAGTGCTTGCAGGG								
c-46	c51605.graph_c0	(TCC)_7_	F: TTCAAGCAAACGCCTTCTTT	206–227	8	8	2.49	0.671	1.276	0.697	5.577
			R: TGAACAGTGGGACCAGATGA								
c-49	c51757.graph_c0	(TGG)_5_	F: GGAAGGGTTAGGGTTATGGG	175–199	7	7	1.97	0.495	0.979	0.606	4.239
			R: TAAAACGGATACAGGGAGCG								
c-52	c52169.graph_c0	(GA)_8_	F: CGTTGTGTGGATTGTTTTCG	216–260	16	16	2.21	0.651	1.380	0.789	12.620
			R: TGGTGGAATCACCACTTCAA								
c-57	c52820.graph_c0	(TTC)_5_	F: GCCCACCTACCTACAACGAA	205–214	4	4	1.82	0.445	0.714	0.428	1.712
			R: TTTCTCCAGAGCTCCCTTCA								
c-59	c53031.graph_c0	(TC)_7_	F: TGATAGGTGCGCAGTTTTTG	236–272	19	19	3.00	0.883	1.467	0.888	16.874
			R: TCCGTACTTGCCGGTTTATC								
c-61	c53112.graph_c1	(GA)_9_	F: GGGAGACTTGTGCGTTTGAT	232–260	14	14	3.01	0.852	1.606	0.850	12.743
			R: AAAGCGTTCTGGTTTGGTAA								
c-69	c54537.graph_c0	(GA)_8_	F: CGAAGTTCTTAAAACCATCA	237–267	15	15	2.79	0.875	1.559	0.881	13.216
			R: CCCACTCCATCTCTGGATTC								
c-73	c54964.graph_c0	(AC)_6_	F: TGAATTAGGGTTTCTCCCCC	328–344	9	9	2.50	0.771	1.455	0.753	6.781
			R: AAAGCCTTCTGGGCTCTCTC								
c-74	c54968.graph_c0	(GA)_7_	F: ATTGCCAATTGTCAGCTCCT	284–292	5	5	2.33	0.666	1.212	0.665	3.324
			R: AACCATGCCCACAAGAAAAG								
c-76	c55159.graph_c2	(AC)_8_	F: GTCATTTCATCCCTGGCTGT	239–265	13	13	2.58	0.701	1.310	0.730	9.493
			R: ACCAAAGTTTCCTGACCCG								
c-77	c55213.graph_c0	(AG)_8_	F: ATCAGTCCTTTTTCGGCCTT	182–204	9	9	2.25	0.592	1.207	0.704	6.339
			R: CACTCTCCCGGATCACATTT								
c-90	c56735.graph_c0	(CT)_8_	F: GCGAAGAAAACAAGTCTCGG	290–304	7	7	1.46	0.688	1.288	0.657	4.597
			R: CTTGTTGCGTGGTCTTGAAA								
c-96	c57507.graph_c0	(CT)_8_	F: GGAGATTGTGGAGAAGCAGC	206–220	8	8	2.17	0.601	1.187	0.668	5.343
			R: AAAAACCCTCCCAAACCATT								
c-97	c57620.graph_c0	(GA)_8_	F: ACCGTTTCATTAACCGCTCC	272–304	15	15	1.94	0.648	1.352	0.778	11.673
			R: AGAAATCACGCCTCTCTCCA								
c-99	c57728.graph_c0	(GTA)_7_	F: CCCATGGCTTTGTCAGATTT	248–281	10	10	2.29	0.802	1.484	0.813	8.134
			R: CCGCTTGTCCCTACACTCAT								
c-100	c58219.graph_c0	(TGG)_6_	F: TCCTTCTCCGCATCATCTCT	290–305	6	6	2.33	0.631	1.170	0.640	3.838
			R: CACGAGTCATCACCAAATCG								
c-112	c59258.graph_c0	(ATC)_6_	F: CCAAAGGCCAAACTGTTGTT	311–359	11	11	2.48	0.721	1.456	0.743	8.175
			R: TCTCAAGATGCTGCTTCCCT								
c-115	c59321.graph_c0	(TTA)_7_	F: TTGCTTCCTTCCTTCCTTGA	200–224	9	9	2.17	0.677	1.274	0.687	5.499
			R: GGTTTGGCCTGGTTTTAGGT								
c-133	c13407.graph_c0	(GAT)_7_	F: ATGAAGCCATTGGTGAGACC	112–145	10	10	2.59	0.688	1.358	0.674	6.736
			R: CCTCCTCTCCTCACAAAACC								
c-146	c29154.graph_c0	(TGG)_6_	F: CCCTACTTTGGGACGACAAG	249–303	17	17	2.72	0.758	1.744	0.753	12.800
			R: CCTCCTTGTTGACGTGGATT								
c-149	c31523.graph_c0	(TTC)_6_	F: TGATCCTACAGAGATGGGGC	190–226	11	11	3.23	0.723	1.430	0.688	7.572
			R: CCCCATGAATCCACAAACAT								
c-150	c34376.graph_c0	(AT)_7_	F: CCCCTCTTCTCTCCTCCATC	228–282	15	15	3.37	0.858	2.133	0.861	12.909
			R: GTTGCTGTTGGGCTTGTTTT								
Total					287	286					
Average					10.63	10.59	2.422	0.689	1.345	0.714	7.969

**Table 5:** Pairwise comparison of Nei’s genetic distance (lower left matrix) and genetic differentiation index (*F*_*ST*_, upper right matrix) among populations and the mean of the genetic distance within populations.

Population	Mean genetic distance	Pop1	Pop2	Pop3	Pop4	Pop5	Pop6	Pop7	Pop8
Pop1	0.369		0.013	0.010	0.008	0.008	0.011	0.012	0.049
Pop2	0.313	0.056		0.015	0.013	0.012	0.016	0.014	0.049
Pop3	0.375	0.053	0.062		0.008	0.010	0.011	0.010	0.055
Pop4	0.363	0.033	0.059	0.040		0.006	0.011	0.008	0.050
Pop5	0.361	0.038	0.059	0.045	0.028		0.010	0.009	0.052
Pop6	0.346	0.061	0.068	0.059	0.043	0.042		0.012	0.045
Pop7	0.372	0.052	0.067	0.056	0.041	0.042	0.048		0.048
Pop8	0.196	0.136	0.145	0.151	0.133	0.136	0.112	0.133	

**Table 6:** Genetic diversity of the eight populations of *S. psammophila*.

Populations	Mean of alleles per locus	Expected heterozygosity H_E_	Shannon-Wiener index	Polymorphism information content (PIC)
POP1	8.111 ± 3.196^a^	0.648 ± 0.147^a^	1.338 ± 0.439^a^	0.649 ± 0.135^a^
POP2	6.815 ± 2.934^a^	0.692 ± 0.116^a^	1.488 ± 0.429^a^	0.602 ± 0.156^a^
POP3	8.111 ± 3.051^a^	0.684 ± 0.121^a^	1.472 ± 0.428^a^	0.652 ± 0.134^a^
POP4	8.074 ± 3.353^a^	0.666 ± 0.127^a^	1.402 ± 0.391^a^	0.647 ± 0.141^a^
POP5	8.074 ± 3.419^a^	0.687 ± 0.128^a^	1.482 ± 0.430^a^	0.642 ± 0.135^a^
POP6	7.222 ± 2.938^a^	0.692 ± 0.118^a^	1.511 ± 0.419^a^	0.625 ± 0.135^a^
POP7	7.852 ± 3.406^a^	0.688 ± 0.117^a^	1.491 ± 0.432^a^	0.644 ± 0.135^a^
POP8	5.519 ± 2.305^b^	0.584 ± 0.161^b^	1.135 ± 0.398^b^	0.523 ± 0.169^b^

Note: ^a^no significant difference at the 0.05 level, ^b^significant difference at the 0.05 level.

**Table 7:** Genetic differentiation among all *S. psammophila* populations and clusters based on AMOVA.

Source of variation	df	Sum of squares	Mean of squares	Percentage of variation (%)
Eight populations				
Among populations	7	511.104	73.015	5.73
Within populations	232	7715.733	31.964	94.27
Total	239	7926.838	33.167	100
Two clusters				
Among clusters	1	306.3267	306.327	18.56
Within clusters	238	7620.511	32.019	81.44
Total	239	7926.838	33.167	100.00
