# Supplementary material for: De novo transcriptome assembly, development of EST-SSR markers and population genetic analyses for the desert biomass willow, Salix psammophila
Source: Sci Rep. 2016 Dec 20;6:39591. doi: 10.1038/srep39591 (PMC5171774; doi:10.1038/srep39591)
Supplement: Supplementary Information [file srep39591-s1.pdf]

Supporting Information

De novo transcriptome assembly, development of EST-SSR markers and population genetic analyses for the desert biomass willow, *Salix psammophila*

Huixia Jia [1, 2], Haifeng Yang [3], Pei Sun [1], Jianbo Li [1], Jin Zhang [1], Yinghua Guo [1], Xiaojiao Han [1], Guosheng Zhang [3], Mengzhu Lu [1, 2] & Jianjun Hu [1, 2, *]

[1] State Key Laboratory of Tree Genetics and Breeding, Key Laboratory of Tree Breeding and Cultivation of the State Forestry Administration, Research Institute of Forestry, Chinese Academy of Forestry, Beijing, 100091, China

[2] Collaborative Innovation Center of Sustainable Forestry in Southern China, Nanjing Forestry University, Nanjing, 210037, China

[3] College of Forestry, Inner Mongolia Agricultural University, Hohhot, 010019, China

[*] Correspondence and requests for materials should be addressed to J.J.H. (email: hujj@caf.ac.cn)

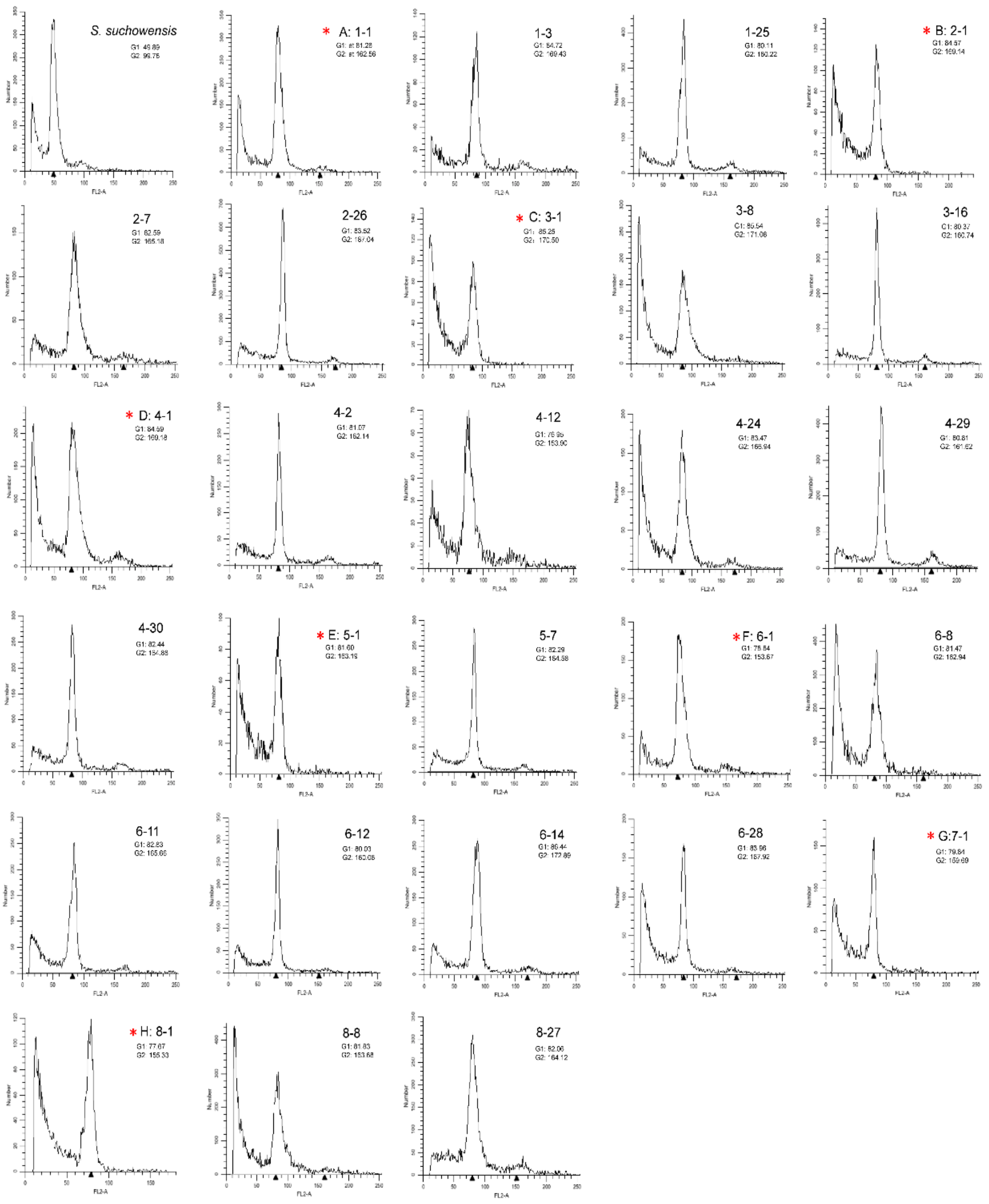

Figure S1. Estimation of the genome size in *S. psammophila*.

The diagram of relative genome size obtained by analyzing the nuclei isolated from young leaf tissues of *S. suchowensis* and 27 *S. psammophila* genets, eight (red star) of which were used for chromosome counts. With its genome size of ~425-429 Mb, *S. suchowensis* served as the internal reference standard. The mean ratio of the G1 peak of *S. psammophila* to that *S. suchowensis* was equal to 1.645, indicating that the genome size of *S. psammophila* is estimated to be ~699-706 Mb. The digits before "-" in the sample names refer to the population number, and digits after "-" refer to the sample number in the corresponding population. For example, the sample name "1-1" means the first sample from Pop1 and "8-27" means the 27th sample from Pop8.

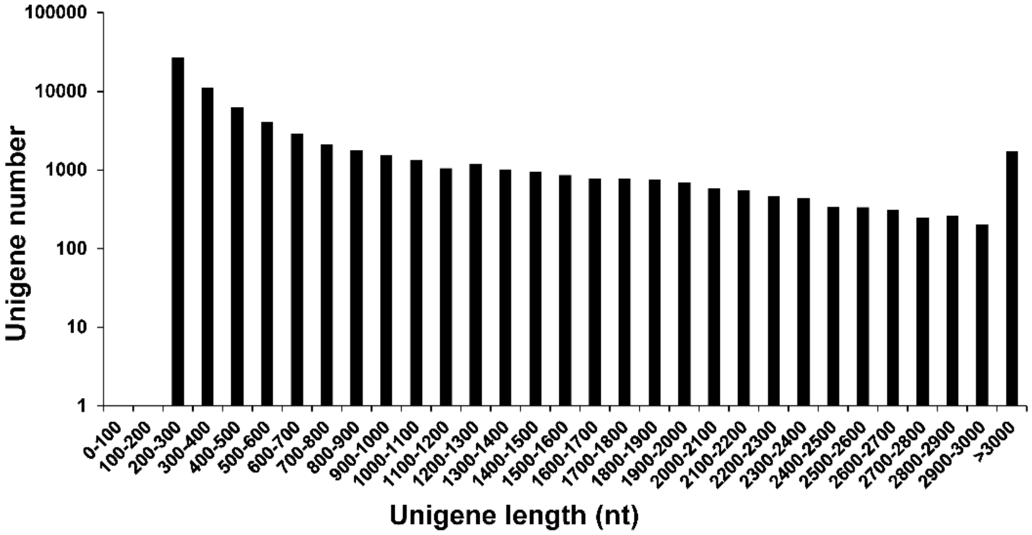

Figure S2. The length distribution of de novo assembled unigenes of *S. psammophila*.

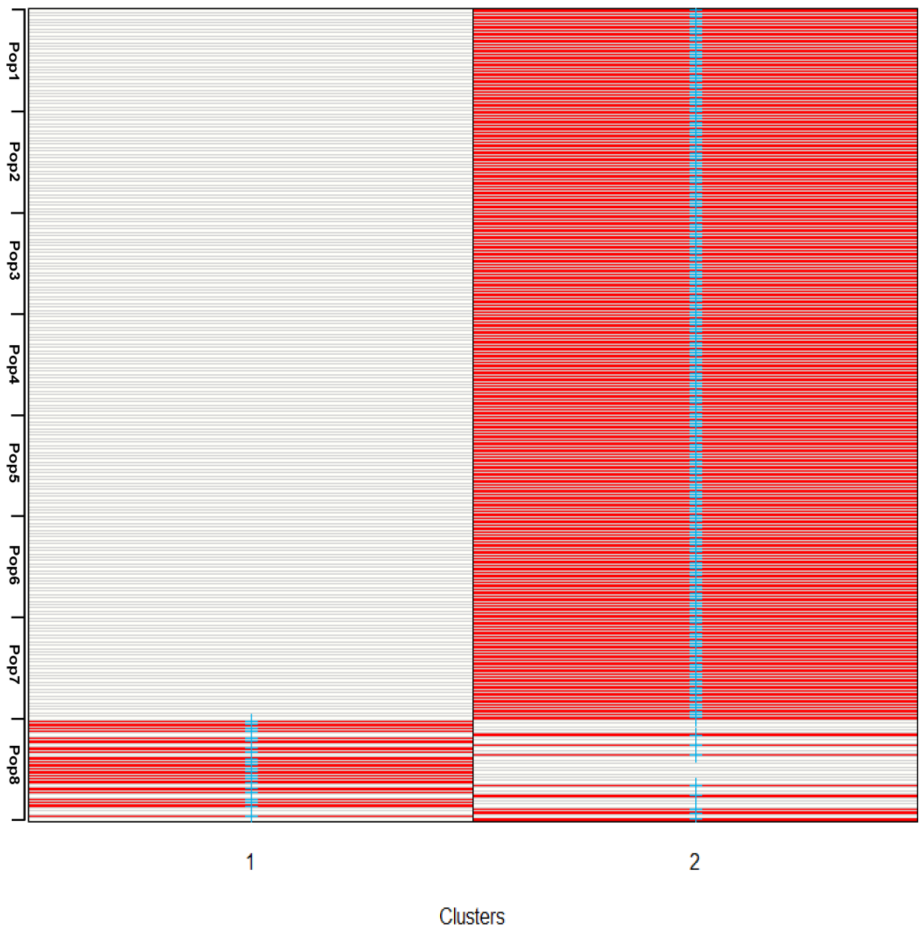

Figure S3. Distribution of 240 *S. psammophila* genets in the two clusters (*K*=2) analysed by the DAPC.

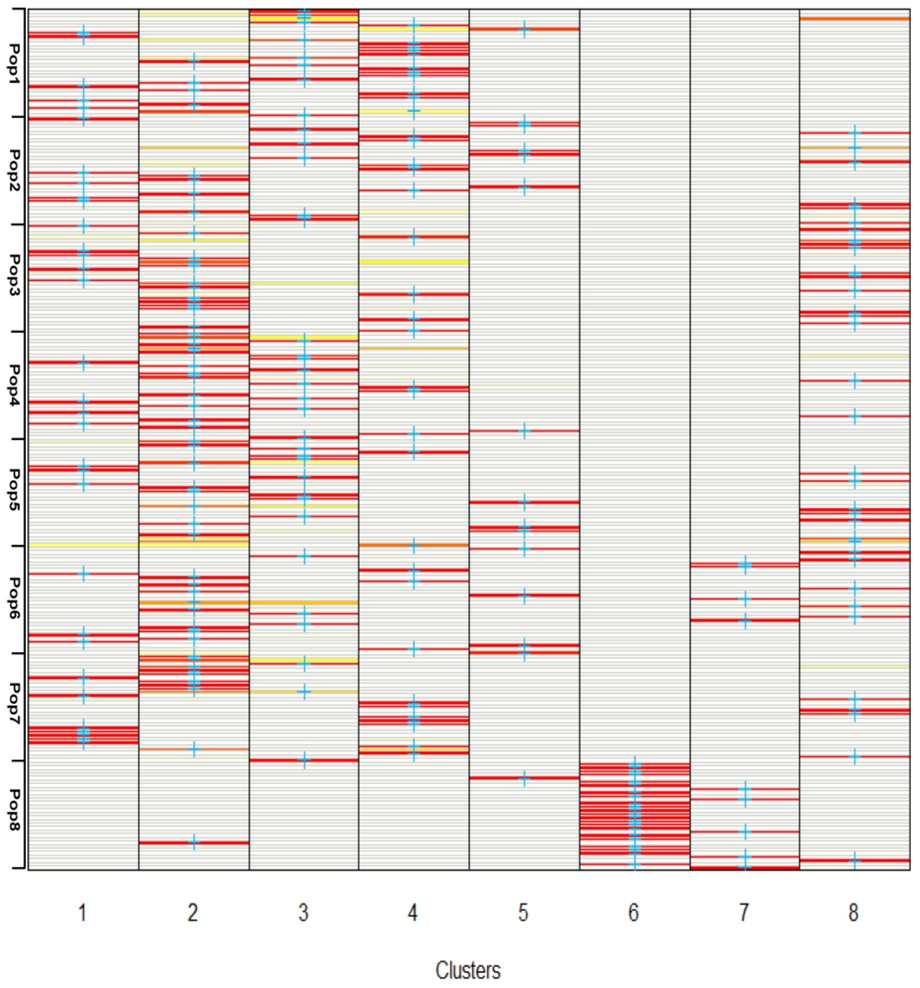

Figure S4. Distribution of 240 *S. psammophila* genets in the eight clusters (*K*=8) analysed by the DAPC.

Supplementary Tables

Table S1. The ratios of the G1 peak of 27 *S. psammophila* genets to *S. suchowensis*

Table S2. GO annotation

Table S3. KOG annotation

Table S4. KEGG annotation

Table S5. Frequencies of different repeat motifs in EST-SSRs

Table S6. Summary frequencies of different SSR repeat motif types

Table S7. Allelic peak ratios calculated for all pairwise allele combinations of 27 loci

Table S8. The genetic distance of the 240 *S. psammophila* genets
